# Phylogenetic meta-analysis reveals system-specific behavioural type–behavioural predictability correlations

**DOI:** 10.1098/rsos.230303

**Published:** 2023-09-06

**Authors:** Gergely Horváth, László Zsolt Garamszegi, Gábor Herczeg

**Affiliations:** ^1^ Department of Systematic Zoology and Ecology, Institute of Biology, ELTE Eötvös Loránd University, Pázmány Péter sétány 1/C, 1117 Budapest, Hungary; ^2^ ELKH-ELTE-MTM Integrative Ecology Research Group, Pázmány Péter sétány 1/C, 1117 Budapest, Hungary; ^3^ Centre for Ecological Research, Institute of Ecology and Botany, Alkotmány u. 2-4, 2163 Vácrátót, Hungary; ^4^ National Laboratory for Health Security, Centre for Ecological Research, Budapest, Hungary

**Keywords:** animal personality, behavioural type, behavioural predictability, covariation, phylogenetic meta-analysis

## Abstract

The biological significance of behavioural predictability (environment-independent within-individual behavioural variation) became accepted recently as an important part of an individual's behavioural strategy besides behavioural type (individual mean behaviour). However, we do not know how behavioural type and predictability evolve. Here, we tested different evolutionary scenarios: (i) the two traits evolve independently (lack of correlations) and (ii) the two traits' evolution is constrained (abundant correlations) due to either (ii/a) proximate constraints (direction of correlations is similar) or (ii/b) local adaptations (direction of correlations is variable). We applied a set of phylogenetic meta-analyses based on 93 effect sizes across 44 vertebrate and invertebrate species, focusing on activity and risk-taking. The general correlation between behavioural type and predictability did not differ from zero. Effect sizes for correlations showed considerable heterogeneity, with both negative and positive correlations occurring. The overall absolute (unsigned) effect size was high (Zr = 0.58), and significantly exceeded the null expectation based on randomized data. Our results support the adaptive scenario: correlations between behavioural type and predictability are abundant in nature, but their direction is variable. We suggest that the evolution of these behavioural components might be constrained in a system-specific way.

## 1. Introduction

Consistent between-individual behavioural variation over time and/or across ecological situations in a population (animal personality) is widespread across various animal taxa (for reviews and meta-analyses see [1–5]). An individual's ‘personality’ is routinely described by the individual's average behaviour (*behavioural type*, e.g. how aggressive an individual is on average), even though less than half of the within-population behavioural variation occurs between individuals [6]. Once the behavioural type and environment-induced individual behavioural plasticity [7,8] are considered in a group (e.g. population), the residual intra-individual variation (rIIV) includes *behavioural predictability* (e.g. how much an individual's aggression varies irrespective of the environment; see [9–11]) and measurement error. Assuming rigorous and standardized behavioural tests, rIIV can be used as a proxy for behavioural predictability when individuals or groups are compared. Interest in behavioural predictability is growing across various disciplines of biology [12–17]. Many studies provided examples of non-random between-individual variation in behavioural predictability across various taxa [9,11,18–25]. The results also imply that between-individual variation in behavioural predictability may be adaptive [16,22,26]. For instance, low predictability may reduce the risk of being captured by predators [18,27,28], while increased predictability may facilitate social interactions [29]. From a neuroendocrinological point of view, previous studies on laboratory rats (*Rattus norvegicus domestica*) [30,31] and strains of rainbow trout (*Oncorhynchus mykiss*) [32] supported the idea that bolder (i.e. more active, aggressive and risk-prone) individuals show lower plasticity and higher predictability compared to their shier conspecifics.

Nevertheless, our understanding of the evolutionary potential of behavioural predictability is still somewhat limited [22,24]. For instance, it is still not clear whether behavioural type generally predicts the level of behavioural predictability. Are bolder individuals more or less predictable than their shier conspecifics? And if so, how general is the behavioural type–behavioural predictability link in nature? These questions are of fundamental importance for understanding the evolution of behaviour, primarily because correlated traits cannot evolve ‘freely’. If behavioural type and behavioural predictability are independent, animal personality can—in theory—emerge not only as a result of natural selection favouring divergent behavioural types within a population, but also as a result of natural selection favouring higher behavioural predictability. On the other hand, selection on trait A will not result in the expected evolutionary change if (i) phenotypic correlations result in indirect selection on trait B (and C, D, etc.) and/or (ii) genetic correlations result in a correlated evolutionary response in trait B (and C, D, etc.) reducing fitness (e.g. [33,34]).

Here, we tested for phenotypic correlations between behavioural type and predictability in a meta-analytic framework. Concerning the evolutionary background of phenotypic correlations, usually two opposing hypotheses, the constraint versus adaptive hypotheses, arise [35,36]. The constraint hypothesis posits that a strong proximate mechanism, like genetic or hormonal background, or developmental linkage generates correlations that can be detected across all (or most) studied taxa. By contrast, the adaptive hypothesis posits that correlations are shaped by ultimate drivers, i.e. divergent natural selection stemming from environmental variation, and therefore predicts that the presence/absence/direction of the correlations are context-specific and variable across taxa. For example, in a predator–prey scenario, occasional mistakes ultimately dictate the survival and fitness of the prey; thus, extreme deviations from the behavioural type would be highly costly for risk-prone individuals (e.g. expressing short latency to emerge from a shelter). This may lead to selection favouring risk-prone individuals being more predictable [37]. Finally, the absence of correlations between behavioural predictability and behavioural type would indicate that these behavioural components are evolving freely, following the independent hypothesis. A recent study showed non-significant tendencies for correlations between behavioural predictability and behavioural type and that some variation exists in the direction of the correlations [37], but they did not test the above hypotheses directly and clear conclusions about the above hypotheses cannot be drawn based on their results.

To test the constraint versus adaptive versus independent hypotheses directly, we conducted a phylogenetic meta-analysis using 93 effect sizes from 58 studies across 44 species of vertebrate and invertebrate taxa, focusing on activity and risk-taking. In our framework, (i) general correlation between behavioural type and behavioural predictability across taxa would support the constraint hypothesis, (ii) lack of general correlation, but variable local correlations would support the adaptive hypothesis, while (iii) lack of both general and local correlations would support the independent hypothesis. We also tested whether various biological and methodological moderator variables (state: age and sex; taxonomical category: vertebrate versus invertebrate; experimental design: environmental conditions, time frame) and behavioural proxies used to quantify behaviour influenced effect sizes.

## 2. Material and methods

### 2.1. Collection of meta-analytical data

For systematic review and meta-analysis, we followed the reporting guidelines of the PRISMA (i.e. Preferred Reporting Items for Systematic Reviews and Meta-Analyses) method [38–40]. For verifying reporting of our study items, we used the PRISMA-EcoEvo guidelines [41]; see electronic supplementary material, table S1. We conducted a literature search using the Web of Science Core Collection database to identify papers on animal personality based on multiple assessments of individual behaviour. We focused on the most frequently studied behavioural traits in the field of personality research (*sensu* Réale *et al*. [42]): activity and risk-taking. Hence, we used a search query for identifying animal personality studies (e.g. ‘animal personalit*’, ‘behavio’ consistency*’, ‘behavio* predictability*’, ‘individual behavio*’, ‘variation*’, ‘strateg*’, ‘repeatabilit*’). For our full search strategy and list of Web of Science indexes covered, see electronic supplementary material, text S1. To obtain the broadest possible coverage, we also conducted cross-reference searches by screening relevant meta-analytic papers [2,37,43,44]. We note that, although we made an effort to find most of the relevant studies, we cannot rule out the possibility that we missed some of them. However, as long as the missed studies were not biased with respect to our main questions, this would not alter our main conclusions.

For the overview and outcomes of our search strategy, see electronic supplementary material, figure S1, for PRISMA diagram. Briefly, we obtained 2325 results from our search of the Web of Science Core Collection and an additional 172 results from the search of relevant meta-analyses. First, duplicates between the databases were removed, leaving 2497 unique works. Next, the titles, abstracts and full texts were examined by GeH to identify potentially relevant primary sources. After the removal of duplicates (note that we considered our publications as duplicates too, since we added our relevant datasets to the analysis at the end of the process; see below), the title and abstract screening of 2410 records and the full-text screening of 1225 studies were conducted by GeH. We were mainly interested in datasets with sufficient sample size, which yield power to test for variation in rIIV (see [37,45]). We considered studies with at least six repeats per individual and a total sample size *N* ≥ 120 (i.e. no. of repeats × no. of individuals), as they should provide an appropriate power in subsequent analyses. In cases where some individuals had fewer than six repeats, we used only the subset of individuals for which there were six repeats, as long as that yielded our criterion on total sample size. Such inclusion criteria are stricter than what available data allowed previously [37]. In cases of studies that used individuals that were subject to manipulation with potential influence on their behaviour (e.g. hormonal or chemical treatment), we used data only from control individuals. Studies conducted over multiple years were considered only if (i) the same individual was tested yearly and/or (ii) the sample size in a given year met our criterion above. Studies testing the same individual repeatedly within a day were considered only if the assays were conducted in different parts of the day (e.g. during the day versus night). Those studies that were selected based on title/abstract, but which subsequently did not meet our criteria when reading the full text (*N* = 1134) are listed in the electronic supplementary material, data S1, along with the reason for their exclusion. Full-text screening identified 91 studies meeting our criteria of inclusion. Full extraction of relevant datasets was possible for 35 studies, while to recover unpublished data, the corresponding authors of the other 56 studies were contacted via a standardized author correspondence email by GeH. As a result, a total of 49 relevant papers encompassing data from 38 species were considered for inclusion in the study. We also included 10 datasets of ours (six published, four unpublished) together with three datasets published by other authors that we collected earlier adding six new species to the database. Altogether, the above approach resulted in 93 effect sizes from 58 studies including 44 species (table 1; note that raw data were identical for three studies), which is considerably larger than what was available a few years ago [37].

**Table 1.** Studies and species included in the meta-analysis.

study	species	common name	class
Barrett *et al*. [46]	*Taeniopygia guttata*	Sunda zebra finch	Aves
Beveridge *et al*. [47]	*Armadillidium vulgare*	common pill bug	Isopoda
Beyts *et al*. [48]	*Engystomops pustulosus*	tungara frog	Amphibia
Biro and Adriaenssens [10]	*Gambusia holbrooki*	eastern mosquitofish	Actinopterygii
Brand *et al*. [49]	*Lampropholis delicata*	delicate skink	Reptilia
Brand *et al*. [50]	*Gambusia holbrooki*	eastern mosquitofish	Actinopterygii
Bridger *et al*. [51]	*Pagurus bernhardus*	marine hermit crab	Malacostraca
Briffa [18]	*Pagurus bernhardus*	marine hermit crab	Malacostraca
Bucklaew and Dochtermann [52]	*Gryllodes sigillatus*	tropical house cricket	Insecta
^a^Carter *et al*. [53]	*Agama planiceps*	Namib rock agama	Reptilia
^a^Carter *et al*. [54]	*Agama planiceps*	Namib rock agama	Reptilia
Cornwell *et al*. [55]	*Littoraria irrorata*	salt marsh periwinkle	Gastropoda
Cornwell *et al*. [56]	*Littoraria irrorata*	salt marsh periwinkle	Gastropoda
Ferderer *et al*. [57]	*Cherax destructor*	common yabby	Malacostraca
Fürtbauer *et al*. [58]	*Gasterosteus aculeatus*	three-spined stickleback	Actinopterygii
Gervais *et al*. [59]	*Capreolus capreolus*	roe deer	Mammalia
Hammond-Tooke *et al*. [60]	*Gobiomorphus cotidianus*	common bully	Actinopterygii
Harris *et al*. [61]	*Rissa tridactyla*	black-legged kittiwake	Aves
Harrison *et al*. [62]	*Thymallus arcticus*	Arctic grayling	Actinopterygii
	*Salvelinus confluentus*	bull trout	Actinopterygii
	*Prosopium williamsoni*	mountain whitefish	Actinopterygii
	*Sander vitreus*	percid walleye	Actinopterygii
	*Oncorhynchus mykiss*	rainbow trout	Actinopterygii
Herczeg *et al*. [63]	*Poecilia reticulata*	Trinidadian guppy	Actinopterygii
Hertel *et al*. [22]	*Ursus arctos*	brown bear	Mammalia
^a^Highcock and Carter [27]	*Agama planiceps*	Namibian rock agama	Reptilia
Holtmann *et al*. [64]	*Prunella modularis*	dunnock	Aves
Holtmann *et al*. [65]	*Prunella modularis*	dunnock	Aves
Holtmann *et al*. [66]	*Prunella modularis*	dunnock	Aves
Horváth *et al*. [67]	*Iberolacerta cyreni*	Carpetan rock lizard	Reptilia
Horváth *et al*. [68]	*Iberolacerta cyreni*	Carpetan rock lizard	Reptilia
Horváth *et al*. [23]	*Armadillidium vulgare*	common pill bug	Isopoda
Horváth *et al*. [69]	*Asellus aquaticus*	common waterlouse	Isopoda
Horváth *et al*. unpublished	*Iberolacerta cyreni*	Carpetan rock lizard	Reptilia
Houslay *et al*. [70]	*Poecilia reticulata*	Trinidadian guppy	Actinopterygii
Ioannou and Dall [71]	*Gasterosteus aculeatus*	three-spined stickleback	Actinopterygii
Kurvers *et al*. [72]	*Poecilia reticulata*	Trinidadian guppy	Actinopterygii
Low *et al*. [73]	*Acheta domesticus*	house cricket	Insecta
Maskrey *et al*. [74]	*Actinia equina*	beadlet sea anemone	Anthozoa
Mathot *et al*. [74]	*Parus major*	great tit	Aves
Mathot *et al*. [75]	*Poecile atricapillus*	black-capped chickadee	Aves
Michelangeli *et al*. [76]	*Tiliqua rugosa*	sleepy lizard	Reptilia
Mitchell and Biro [25]	*Danio rerio*	zebrafish	Actinopterygii
Mitchell *et al*. [77]	*Poecilia reticulata*	Trinidadian guppy	Actinopterygii
Mitchell *et al*. [78]	*Poecilia reticulata*	Trinidadian guppy	Actinopterygii
O'Dea *et al*. [11]	*Danio rerio*	zebrafish	Actinopterygii
Ólafsdottir and Magellan [79]	*Gasterosteus aculeatus*	three-spined stickleback	Actinopterygii
Orf *et al*. unpublished	*Rana dalmatina*	agile frog	Amphibia
Pezner *et al*. [80]	*Spirobranchus giganteus*	Christmas tree worm	Polychaeta
Polverino *et al*. [81]	*Gambusia affinis*	western mosquitofish	Actinopterygii
Prentice *et al*. [82]	*Poecilia reticulata*	Trinidadian guppy	Actinopterygii
Raffard *et al*. [83]	*Procambarus clarkii*	red swamp crayfish	Malacostraca
Rotics *et al*. [84]	*Ciconia ciconia*	white stork	Aves
Sakai [85]	*Lepidodactylus lugubris*	mourning gecko	Reptilia
Santostefano *et al*. [86]	*Gryllus campestris*	field cricket	Insecta
Sos *et al*. unpublished	*Zootoca vivipara*	common lizard	Reptila
Stamps *et al*. [9]	*Pagurus bernhardus*	marine hermit crab	Malacostraca
Sztruhala *et al*. unpublished	*Armadillidiuam vulgare*	common pill bug	Isopoda
Tan *et al*. [87]	*Bufo gargarizans*	Asiatic toad	Amphibia
Thoré *et al*. [88]	*Nothobranchius furzeri*	turquoise killifish	Actinopterygii
Toscano *et al*. [89]	*Planorbella trivolvis*	marsh rams-horn	Gastropoda
	*Physa acuta*	European physa	Gastropoda
Urszán *et al*. [90]	*Rana dalmatina*	agile frog	Amphibia
Vallon *et al*. [91]	*Pomatoschistus microps*	common goby	Actinopterygii
Wexler *et al*. [92]	*Tribolium castaneum*	red flour beetle	Insecta

^a^Data are identical for these studies.

### 2.2. Moderators

Both biological and methodological differences between studies are expected to affect the variation of association between components of behavioural strategy; thus, we accounted for these potential sources of heterogeneity by extracting and examining the effect of various moderator variables. The following variables were extracted from each study as methodological predictors: (i) environmental conditions during the behavioural tests (i.e. laboratory, natural and semi-natural) and (ii) time context in which individual behaviour was repeatedly tested (approximately one week, 2–3 weeks, approximately one month, several months, one or more years). Biological predictors included (iii) sex (female, male or both (in the case of one study, sex was not applicable)), (iv) age (juvenile, adult or both (in the case of one study, age was not applicable)), (v) behavioural trait (activity or risk-taking) and (vi) taxa (invertebrate or vertebrate).

### 2.3. Statistical methods

For each study that satisfied our inclusion criteria, raw data that we received from authors or extracted from either supplementary materials or digital repositories (i.e. Dryad, Figshare, OSF) were re-analysed to estimate behavioural type and behavioural predictability and calculate correlations between them. Importantly, as the research questions of the studies considered here differed fundamentally from ours, it is rather unlikely that a non-significant association between behavioural type and behavioural predictability occurs with a higher chance in non-published works than in publications we examined, but still, our results should be interpreted with care. We chose to run the analyses on the raw data because this approach introduces less heterogeneity than extracting effect sizes from complex statistical analyses presented in the source papers, also allowing us to run double hierarchical mixed models (DHLMMs). Datasets were reanalysed using the R package *brms* [93,94] based on the Bayesian software Stan (version 2.26.1 [95]). The main advantage of DHLMMs above multi-step (stats-on-stats) approaches is that they allow for the simultaneous estimation of a ‘mean model’ and a ‘residual model’, thus errors that are associated with a parameter estimation in one model are considered in the other model. The ‘mean model’ estimates whether individuals differ in their mean expression of behaviour (i.e. behavioural type), while the ‘residual model’ estimates whether they differ in rIIV around this behavioural mean (i.e. behavioural predictability). Our R code and output are provided as electronic supplementary material. Each dataset was standardized (mean = 0, SD = 1) and analysed using a Gaussian distribution; this transformation does not affect the parameters of interest but aids model fitting. In our models, we accounted for contextual and/or temporal plasticity by including random slope effects for time and/or context where it was applicable.

We used weakly informative normal priors (N(0,5)) for fixed effects, half-normal priors (N(0,5)) for random effects, and an LKJ(1)–correlation prior for the correlation of random effects [96]. We ran four chains to evaluate convergence, which was run for 4000 iterations, with a warmup of 1000 iterations and a thinning interval of 4. From each model, we extracted the correlation of rIIV (a random intercept in the residual model) with the mean level trait (at mean-centred random slopes where they were present). As any distribution that is limited by zero will have a positive mean-variance relationship, behavioural traits measured on a ratio scale (i.e. latency variables, total distance, number of movements) are expected to show a positive relationship between behavioural type and behavioural predictability as a statistical artefact. To resolve this problem, the aforementioned behavioural traits were analysed on the log scale.

Extracted correlation coefficients (*r*) were transformed to Fisher's normalized correlation coefficients (Zr) [97] to normalize the data for meta-analysis (equation (2.1)). Estimates with 95% credibility intervals (CIs) that do not cross zero are inferred to represent real effects at *α* = 0.05.

2.1 $$\mathrm{Zr}=\frac{1}{2}\ln\left(\frac{r+1}{r-1}\right).$$

As some behavioural traits were interpreted as latency variables in our dataset (i.e. time spent under a shelter or in cover, time to enter a nest, time to restart activity, flight initiation distance), small Zr values indicate high risk-taking/activity in these cases. These Zr values were multiplied by −1 to interpret all data on the same conceptual scale.

The meta-analytic approach assumes that between-observation variance caused by sampling error could be approximated by squared standard error; nevertheless, sampling variance for low-precision estimates (i.e. a low number of individuals sampled or a low number of repeats) is higher. This may lead to (i) low-precision studies having too much weight, (ii) some amount of biological variation being overestimated and (iii) mean effect size potentially being biased if publication bias is more likely among low-precision studies. We recorded the number of individuals measured in a study (*N*_individual_) and the number of repeats per individual (*N*_repeats_) to calculate the variance in effect size, following Holtmann *et al*. [98] (equation (2.2)):

2.2 $$\mathrm{var}=\;\frac{N_{\mathrm{repeats}}}{2\;\times \;(N_{\mathrm{individual}}-2)\;\;\times \;(N_{\mathrm{repeats}}-1)}\;.$$

To account for the non-independence of effect sizes (see [99] for details), we applied multi-level meta-analytic models to estimate effect sizes in the case of each behavioural trait (i.e. activity and risk-taking) separately, estimating their global effect sizes. Preliminary descriptive analyses revealed a strong association between study identity and species, i.e. all but one studies focused on a single species, while some species were targeted by multiple studies (*Poecilia reticulata*, *N* = 6; *Iberolacerta cyreni*, *N* = 3; *Pagurus bernhardus*, *N* = 3; *Armadillidium vulgare*, *N* = 3; *Gasterosteus aculeatus*, *N* = 3; *Prunella modularis*, *N* = 3; *Rana dalmatina*, *N* = 2; *Danio rerio*, *N* = 2; *Gambusia holbrooki*, *N* = 2; *Littoraria irrorata*, *N* = 2; table 1). According to deviance information criterion (DIC) values, adding species identity and effect size identity along with study identity and phylogeny as random effects greatly improved the model fit (DIC = −72.29 versus 203.73); thus, both variables were included in the final models.

We obtained information on the phylogenetic history of species based on published phylogenies available through the Open Tree of Life [100] via the *rotl* package [101]. Taxon names were matched to records in the Open Tree Taxonomy to obtain relationships between species. Due to the diversity of species in our meta-analyses, accurate estimation of branch lengths was not possible; thus, we computed branch lengths based on topology (see electronic supplementary material, figure S2) using the *ape* package [102] in R 4.2.2. [103]. Phylogenetic heritability or phylogenetic signal (H^2^) was calculated as the proportion of total variance in Zr that can be explained by phylogenetic variance [104], equivalent to Pagel's *λ* [105]. H^2^ = 0 indicates no phylogenetic relatedness among effect sizes [106].

Meta-analyses were conducted with generalized linear mixed models (GLMMs) with Markov chain Monte Carlo techniques using the R package MCMCglmm [107]. All models were fitted using an uninformative inverse gamma prior for study identity and phylogeny as random effects and were checked for sensitivity to prior specification and convergence across independent model runs (following Wilson *et al*. [108]). We first ran intercept-only mixed models (with random effects) to determine the mean effect size across all studies. To estimate heterogeneity of effect sizes, we used I^2^ statistics [109–111] modified for multilevel meta-analytic models [99]: total heterogeneity (I^2^_total_) was partitioned into phylogenetic variance ($I_{\mathrm{phylogeny}}^{2}$), study ID variance ($I_{\mathrm{study}}^{2}$) and residual variance ($I_{\mathrm{residual}}^{2}$). Low, moderate and high heterogeneities refer to I^2^ of 25%, 50% and 75%, respectively [109]. Next, we constructed a series of meta-regression models to identify the most important moderators (see above) [99]. As our sample size was somewhat limited, we chose to avoid complex models; instead, we conducted fixed-effect mixed models to estimate the mean effect size for each moderator separately [112]. Model diagnostics based on variance inflation factor (VIF) indicated no evidence for multicollinearity (all VIFs < 4). Models with categorical moderators were run without the intercept to test each trait against no effect. Parameter estimates were based on posterior means.

We also tested whether the observed absolute (unsigned) mean effect sizes significantly deviate from what is expected under the null hypothesis of no relationship between mean behaviour and rIIV by generating a null expectation based on simulations. Basically, for each source study, we generated random numbers with the original sample size for each Zr, from which we calculated absolute effect sizes. Then, we implemented the meta-analyses on the simulated effect sizes (all unsigned) to calculate absolute effect sizes for the null hypothesis by relying on the same model design as for the real data [113]. The procedure was repeated 1000 times to get a null distribution, against which the significance of the absolute effect sizes of the real datasets was assessed (see also our R code in the electronic supplementary material).

## 3. Results

**Figure 1.**
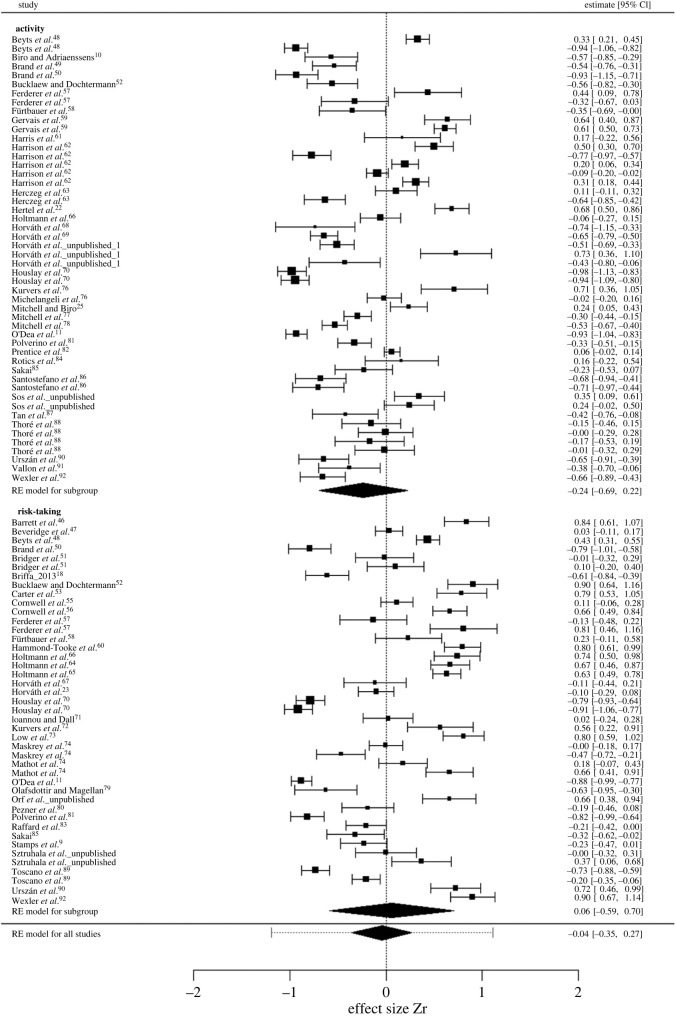
Forest plot of original effect sizes for raw behavioural type–behavioural predictability correlations across studies. Effect sizes and associated variances were extracted from each study. Black polygons indicate means from the random-effect (RE) model for activity and risk-taking presented separately and all data combined, and error bars represent the meta-analytic variance *N*_repeats_/(2 × (*N*_individual_ − 2) × (*N*_repeats_ – 1)).

**Figure 2.**
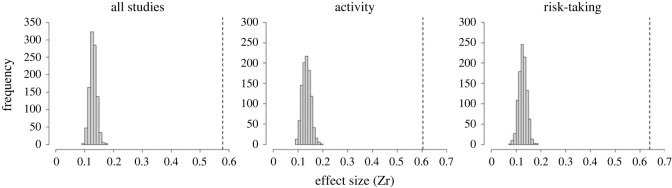
Absolute effect sizes for correlation between behavioural type and behavioural predictability for all data combined, activity, and risk-taking. Observed absolute effect sizes (broken line) as compared to the simulated absolute effect sizes under null expectation (histograms; see Material and methods for the details) are shown.

**Figure 3.**
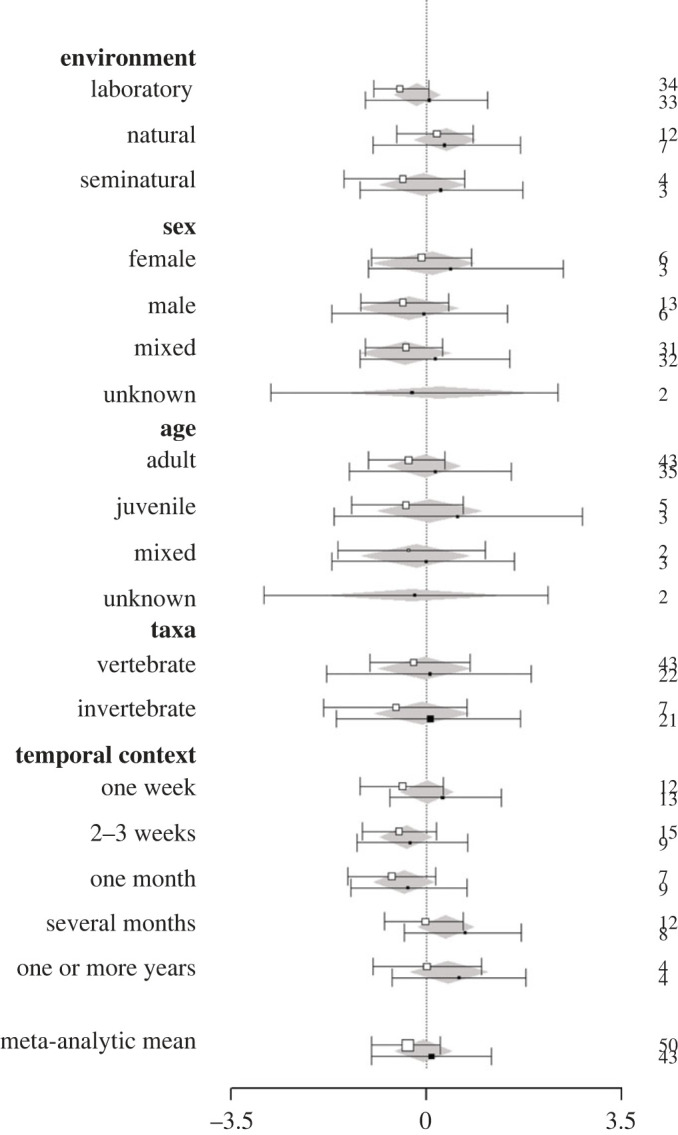
Effect of moderators on strength of behavioural type–behavioural predictability correlations across studies. Boxes show the mean posterior estimates from the model, and error bars represent the 95% highest posterior density (HPD) intervals. Numbers on the right-hand side of each panel indicate the number of effect sizes in each subgroup. White boxes indicate model estimates from activity data, black boxes are from risk-taking data, and grey polygons indicate estimates from models using all behavioural data. The meta-analytic mean is from the intercept-only model run with species identity, effect size identity along with study identity and phylogeny as random effects.

Considering all studies, we found no significant general correlation between behavioural type and predictability (mean Zr (95% CI) = −0.05 (−0.56–0.45), *N* = 93). The total heterogeneity in effect size was large (98.68%; see also table 1), phylogenetic differences explained only a low amount of it ($I_{\mathrm{phylogeny}}^{2}=21.48\text{\%}$), and comparably, heterogeneity among studies was rather low as well ($I_{\mathrm{study}}^{2}=16.83\text{\%}$). As 66% of the effect sizes were significant (figure 1 shows effect sizes on raw correlation coefficients and results from random-effect models accounting for study ID, effect size ID, species ID and phylogeny), the lack of general correlation (the mean effect size not differing from zero) was most likely not a result of lack of correlations in different studies, but rather the difference in their directions. This is confirmed by the fact that the overall mean absolute effect size was significantly larger than the one calculated under the null expectation (Zr [observed] = 0.58 versus Zr [null] = 0.129, *p* *<* 0.001) (figure 2*a*). While heterogeneity was mostly explained by our random effects, substantial residual variation persisted ($I_{\mathrm{residual}}^{2}=16.28\text{\%}$; table 2). We ran univariate models to identify moderators potentially explaining residual heterogeneity, and while none of the separate one-predictor models indicated significance for any of the moderator variables (electronic supplementary material, table S2), adding ‘sex’ as moderator variable reduced residual heterogeneity substantially (electronic supplementary material, table S5). Also, there was significant contrast between activity and risk-taking behaviours (*p* < 0.001; see electronic supplementary material, table S8): behavioural type–predictability correlation showed a negative trend in activity (figure 1 and figure 3). Further, there was a significant contrast between studies conducted under controlled conditions (i.e. in the laboratory) and in the natural habitat (*p* = 0.026; figure 3 and electronic supplementary material, table S8): behavioural type–predictability correlation estimates showed a negative trend for studies conducted in the laboratory. Also, we found significant contrast between several categories of the variable ‘temporal contexts’ (figure 3 and electronic supplementary material, table S8): correlation estimates showed a positive trend in long-term studies (i.e. conducted over several months or years). Phylogenetic heritability was rather low, but present (mean H^2^ = 21.73 (0–52.46); mode H^2^ = 0.2).

**Table 2.** Heterogeneity estimates and deviance information criteria (DIC) for a set of random-effect-only meta-analytical models for correlation between behavioural type and behavioural predictability. The total I^2^ (and the 95% highest posterior density (HPD) intervals) is the sum of all variance components. The mode total variance is shown for comparison (where similar values indicate stable models). Phylogenetic heritability (H^2^) is the proportion of variance that can be explained by phylogenetic variance [104]. The final random-effect model used in subsequent analyses is highlighted in bold.

data set	*N*	model ID	DIC	$I_{\mathrm{residual}}^{2}\;(\mathrm{HPD})$	$I_{\mathrm{phylogeny}}^{2}\;(\mathrm{HPD})$	$I_{\mathrm{species}}^{2}\;(\mathrm{HPD})$	$I_{\mathrm{study}}^{2}\;(\mathrm{HPD})$	$I_{\mathrm{ES}}^{2}\;(\mathrm{HPD})$	$I_{\mathrm{mean}\;\mathrm{total}}^{2}\;(\mathrm{HPD})$	mode total I^2^	mean H^2^ (HPD)	mode H^2^
all	93	Model.null	221.87	98.41 (97.98–98.9)					98.41 (97.98–98.9)	98.45		
		**Model.full**	**−72.29**	**16.28** **(0.02–58.68)**	**21.47** **(1.41 × 10^−5^–52.04)**	**4.28** **(2.29 × 10^−6^–15.86)**	**16.83** **(7.54 × 10^−5^–36.95)**	**39.81** **(0.0001–73.71)**	**98.68** **(98.12–99.27)**	**98.68**	**21.73** **(0–52.46)**	**0.2**
activity	50	Model.null	113.11	98.27 (97.56–98.92)					98.27 (97.56–98.92)	98.5		
		**Model.full**	**−42.09**	**14.9** **(0.03–56.03)**	**22.25** **(1.29 × 10^−6^–60.41)**	**4.53** **(3.79 × 10^−8^–17.19)**	**21.44** **(3.69 × 10^−6^–51.33)**	**35.62** **(0.0002–75.79)**	**98.74** **(98–99.45)**	**99.66**	**22.48** **(0–60.95)**	**0.38**
risk-taking	43	Model.null	104.79	98.25 (97.51–98.95)					98.25 (97.51–98.95)	98.34		
		**Model.full**	**−43.59**	**6.41** **(0.005–26.07)**	**59.22** **(11.56–95.57)**	**7.89** **(3.42 × −10^−7^–33.03)**	**11.22** **(2.74 × −10^−8^–37.68)**	**14.32** **(9.31 × −10^−7^–41.01)**	**99.06** **(98.25–99.77)**	**99.15**	**59.7** **(11.74–95.72)**	**75.14**

Separate examination of activity and risk-taking data revealed no significant general correlation between behavioural type and behavioural predictability (activity: Zr = −0.33 (−0.98–0.25), *N* = 50; risk-taking: Zr = 0.09 (−0.99–1.16), *N* = 43). Total heterogeneity was high for both activity (98.68%) and risk-taking (99.06%). In the case of activity, differences across phylogeny (activity $I_{\mathrm{phylogeny}}^{2}:\;22.25\text{\%}$) and studies (activity $I_{\mathrm{study}}^{2}:\;21.44\text{\%}$) explained a similar amount of total heterogeneity, while for risk-taking, phylogeny alone was responsible for most of it (risk-taking $I_{\mathrm{phylogeny}}^{2}:\;59.22\text{\%}$), heterogeneity across studies was rather low (risk-taking $I_{\mathrm{study}}^{2}:\;11.22\text{\%}$). Similar to the pooled sample, the percentage of significant effect sizes in both behavioural traits (activity: 70%; risk-taking: 63%; figure 1) suggest substantial interspecific variation in the strength and direction of the correlations across studies. This is again confirmed by our null expectation models (activity: Zr [observed] = 0.6 versus Zr [null] = 0.134, *p* < 0.001; risk-taking: Zr [observed] = 0.64 versus Zr [null] = 0.127, *p* < 0.001; figure 2).

Substantial residual variance persisted for activity (14.9%), while in the case of risk-taking, it was rather low (6.4%). When testing categorical level differences in behavioural type–behavioural predictability correlations using univariate models, we found no significant moderator effect in the case of activity or risk-taking (see electronic supplementary material, tables S6 and S7). Nevertheless, we found significant contrast between categories of different variables. In the case of activity, the variable ‘environment’ showed significant contrast between the categories laboratory and natural (*p* = 0.026): similar to the result found in the pooled sample, correlation estimates showed a positive tendency for studies conducted under natural conditions. In the case of risk-taking, various categories of the variable ‘temporal context’ showed significant contrasts: estimates from studies conducted on the long term showed a positive trend, as well as short-term ones. We found that residual heterogeneity could not be reduced substantially by adding any of the moderator variables to the full model for neither of the behaviours (see electronic supplementary material, tables S6 and S7). Phylogenetic heritability in activity was rather low, but present (mean H^2^ = 22.48 (0–60.95); mode H^2^ = 0.38), while it was rather high in the case of risk-taking (mean H^2^ = 59.7 (11.74–95.72); mode H^2^ = 75.14).

## 4. Discussion

Most importantly, we found that the general correlation between behavioural type and behavioural predictability did not differ significantly from zero, with no difference between vertebrates and invertebrates or the studied behaviours. Based on this result, we rejected the constraint hypotheses. In other words, we found no support for the idea that a general proximate (genetic, developmental, physiological) constraint forces behavioural type and predictability to covary on the phenotypic level. Similar to our findings reported here, a recent meta-analysis from Mitchell *et al.* [37] found no general correlation between behavioural type and behavioural predictability in risk-taking and activity across vertebrate and invertebrate taxa, but a slight, nonsignificant tendency for risk-prone individuals being less predictable, while more active ones more predictable. None of the moderator variables had significant effects on the correlation between these behavioural components, although we could detect significant contrasts among the different levels of the moderator variable ‘behaviour’, ‘environment’ as well as ‘temporal context’.

Even though we can reject the constraint hypothesis, we should still decide whether the patterns support the adaptive hypothesis (strength and direction of correlations depend on the studied system) or whether the studied traits are independent (correlations nearly or fully absent). Several theoretical and empirical studies argued that an individual's behavioural type and behavioural predictability should covary, either positively or negatively [9,16]. Most recently, Mitchell *et al*. [37] suggested that one possible explanation behind such patterns might be that unreliable cues from the environment may increase behavioural predictability, and if individuals differ in their ability to process environmental information, e.g. due to variation in exploration tendencies or sensory system, this might lead not just to differences in behavioural predictability, but to covariation between behavioural predictability and behavioural type. However, as was mentioned above, only a handful of studies tested whether behavioural type constrains behavioural predictability empirically, with results indicating no clear pattern and invoking several potential biological explanations [16].

In their pioneering work, Stamps *et al*. [9] found that behavioural predictability of risk-taking showed a weak positive correlation with risk-taking behavioural type in hermit crabs (*Pagurus bernhardus*), where risk-prone individuals emerging quicker from their shells after disturbance were more predictable, plausibly as a result of constraints in the individuals’ ability to estimate time intervals. Briffa *et al*. [19] reported similar patterns from the same species, and more recently, studies on guppy (*Poecilia reticulata*) [77] and three-spined stickleback (*Gasetrosteus aculeatus*) [24] also found that high levels of activity and risk-taking are coupled with high predictability. On the other hand, Highcock & Carter [27] reported a negative link between risk-taking behavioural type and predictability in Namibian rock agama (*Agama planiceps*), as risk-prone individuals were less predictable. Similarly, Mitchell & Biro [25] found more active wild-type zebrafish (*Danio rerio*) to be less predictable than their less active conspecifics. In line with the above contradicting results, we found that the overall mean absolute effect size was higher than predicted by the null model for the behavioural type–behavioural predictability correlation. This shows that the lack of a general correlation between behavioural type and behavioural predictability is a result of the considerable interspecific variation in the strength and direction of the correlations across studies. This large heterogeneity indicates that particular effects in different studies represent different biological backgrounds and the correlation between behavioural type and behavioural predictability is affected by different local selective forces. Hence, local adaptation might be the main driver behind the emerging behavioural type–behavioural predictability correlations, rather than physiological, developmental or environmental constraints.

We detected moderate–high phylogenetic inertia for correlations between behavioural type and behavioural predictability across all data and separately examined behavioural traits. However, our results are likely influenced by the lack of statistical power in some taxa. We have to note that our phylogenetic tree was built based on a limited number of species, and more importantly, it was strictly topological and did not contain branch lengths. Thus, the level of phylogenetic inertia detected in our analyses should be treated as a preliminary result. We advise conducting similar syntheses in more restricted, closely related invertebrate and vertebrate taxonomic groups, with well-known phylogenetic relationships (for instance, birds [114,115] and fishes [116]) to reveal whether covariation between components of behavioural strategy is phylogenetically conservative when data become available. Further, intraspecific studies comparing populations adapted to different environments would provide further insight into the evolution of the link between behavioural type and behavioural predictability.

## 5. Conclusion

In summary, our results did not indicate a general correlation between behavioural predictability and behavioural type. We suggest that the lack of general correlation is rather shaped by local selective forces resulting in opposing correlations than an overall lack of correlations. Hence, the two traits are often (in 66% of assessed cases) not independent, but their relationship is species-specific. Empirical studies using cut-edge methodology to reveal covariation between components of between- and within-individual behavioural variation started to accumulate lately [9,10,22–27,47,77,117], but the evolutionary analysis of behavioural predictability is still in the pioneering stage. We hope that our synthesis highlights the need for additional empirical research applying advanced experimental design (>6 number of individual repeated assays coupled with robust statistical methods). Importantly, studies on behavioural predictability need to seek links with underlying proximate mechanisms (e.g. physiological, genetic) and test their associations with fitness. We also propose to conduct studies spanning over several seasons or years, or assaying individual behavioural responses in different environments to assess behavioural type, behavioural predictability and behavioural plasticity parallel (for examples, see [19,23,74,90]). Such criteria pose serious difficulties to research on wild populations; however, several recent examples demonstrate that bio-logging may be an especially powerful tool to overcome such challenges [22,62,84,118].

## Data Availability

Literature search data, detailed code for the analyses and model outputs can be found in the Open Science Framework (OSF): https://osf.io/n83ka/. The data are provided in electronic supplementary material [119].
